# The genomic basis of cichlid fish adaptation within the deepwater “twilight zone” of Lake Malawi

**DOI:** 10.1002/evl3.20

**Published:** 2017-08-29

**Authors:** Christoph Hahn, Martin J Genner, George F Turner, Domino A Joyce

**Affiliations:** ^1^ Evolutionary and Environmental Genomics Group (@EvoHull), School of Environmental Sciences University of Hull Hull HU5 7RX United Kingdom; ^2^ Institute of Zoology University of Graz A‐8010 Graz Austria; ^3^ School of Biological Sciences University of Bristol Bristol Life Sciences Building, 24 Tyndall Avenue Bristol BS8 1TQ United Kingdom; ^4^ School of Biological Sciences Bangor University Bangor Gwynedd LL57 2UW Wales United Kingdom

**Keywords:** cichlid, hemoglobin, Root effect, sensory drive, supergene

## Abstract

Deepwater environments are characterized by low levels of available light at narrow spectra, great hydrostatic pressure, and low levels of dissolved oxygen—conditions predicted to exert highly specific selection pressures. In Lake Malawi over 800 cichlid species have evolved, and this adaptive radiation extends into the “twilight zone” below 50 m. We use population‐level RAD‐seq data to investigate whether four endemic deepwater species (*Diplotaxodon* spp.) have experienced divergent selection within this environment. We identify candidate genes including regulators of photoreceptor function, photopigments, lens morphology, and haemoglobin, many not previously implicated in cichlid adaptive radiations. Colocalization of functionally linked genes suggests coadapted “supergene” complexes. Comparisons of *Diplotaxodon* to the broader Lake Malawi radiation using genome resequencing data revealed functional substitutions and signatures of positive selection in candidate genes. Our data provide unique insights into genomic adaptation within deepwater habitats, and suggest genome‐level specialization for life at depth as an important process in cichlid radiation.

Deepwater environments pose an array of physiological and ecological challenges to the organisms that inhabit them. Below 50 m, high hydrostatic pressure, reduced levels of dissolved oxygen, and a lack of ambient light will all produce characteristic selection pressures (Sébert and Macdonald 1993; Morita 2010). Evolutionary adaptation of species to these challenges should be possible to detect at the genomic level, and yet surprisingly few studies have addressed this. In the context of ecological speciation and adaptive radiation, divergence along depth gradients is associated with the evolution of reproductive isolation in many marine (Wilson and Hessler 1987; Jennings et al. 2013; Brown and Thatje 2014; Shum et al. 2014) and freshwater species groups. Specifically, fish species within adaptive radiations of the freshwater Lakes Baikal, Tanganyika, and Malawi differ extensively in the depth ranges that they occupy. Thus, investigating the genomic regions involved should provide powerful insights into the rapid ecological adaptation in these species, including physiological and ecological characteristics that have been subject to divergent selection.

High hydrostatic pressure associated with depth affects multiple biological processes, from the activity of macromolecular protein assemblages such as tubulin and actin, to cellular processes such as osmoregulation and actin potential transmission in nervous cells (Somero 1992; Pradillon and Gaill 2006). As a consequence, pressure can affect the nervous system, cardiac function, and membrane transport systems relatively quickly, whereas other systems are more resilient to change (Brauer and Torok 1984). Coupled with the increase in pressure is a decline in the spectral range and intensity of ambient light (Bowmaker and Hunt 2006). In aquatic systems light intensity decreases exponentially with water depth (Tyler 2003) and in clear water the long wavelength portion of the visible light spectrum (red light) is increasingly attenuated, shifting the spectral median toward short wavelength, monochromatic (blue) “twilight” conditions (Von der Emde et al. 2012) in deepwater environments. In marine mesopelagic fishes perhaps the most recognized morphological adaptation of the visual system for life at increased depth is eye enlargement, which accommodates the reduced light intensity by increasing the chance of photon capture (Marshall and Marshall 1954; de Busserolles et al. 2013). In addition, shifts in the relative abundance of rods and cones in the retina have been associated with habitat depth (Landgren et al. 2014; Hunt et al. 2015); in vertebrates, cones mediate photopic vision under bright light conditions, whereas rods contain specialized pigments for scotopic vision under dim light conditions (Fernald 1988).

Comparative analyses of the light absorption spectra of photopigments in different species have firmly established the role of “spectral tuning” in sensory adaptations, that is, shifts in the maximum spectral sensitivity of photopigments toward the peak wavelength of the available light (Wang et al. 2011; Nakamura et al. 2013; Carleton 2014; Malinsky et al. 2015). Differential expression and alterations in amino acid sequences of opsin genes have been identified as the underlying molecular mechanisms for spectral shifts (Carleton 2009; Nakamura et al. 2013; Cortesi et al. 2015; Malinsky et al. 2015). In the context of deepwater adaptations, a range of genomic modifications affecting opsin genes, which code for the rod and cone pigments, have been implicated in spectral tuning in marine mesopelagic (Wang et al. 2011; Nakamura et al. 2013; Shum et al. 2014), and freshwater fishes, including the Lake Baikal sculpins (Hunt et al. 1997) and deepwater cichlids of the East African great lakes (Sugawara et al. 2005). In this study we consider the adaptations of Lake Malawi's twilight zone (50–220 m) cichlids in more detail. This region is inhabited by members of an endemic deepwater haplochromine cichlid lineage that includes approximately 20 species of *Diplotaxodon*, plus the closely related *Pallidochromis tokolosh*, all of which are zooplanktivorous or piscivorous (Turner et al. 2004). Interspecific divergence must have happened since Lake Malawi achieved deepwater conditions in the last five million years, or even more recently given evidence that the lake has been dry or shallow for much of its history (Delvaux 1993; Ivory et al. 2016). Sympatric species in the lineage often differ in male monochromatic nuptial colour and morphological traits, including eye size (Genner et al. 2007). Currently, little empirical data on species specific depth distributions of *Diplotaxodon* are available. Survey catch records indicate that species within the *D. macrops* complex are typically found between 100 and 220 m during the day, with peak abundance at 220 m. By contrast species in the *D. limnothrissa* complex are typically found between 30 and 220 m during the day, with peak abundance at 60 m (Thompson and Allison 1996). Representatives of both species complexes undergo diurnal vertical migrations to shallower waters at night (Thompson and Allison 1996). Previous work also indicates that *Diplotaxodon* species can differ in their breeding locations, which covary with differences in water depth (Genner et al. 2010).

In this study we use population‐level genome‐wide single nucleotide polymorphism (SNP) data to characterize the genomic divergence and signatures of selection between four sympatric *Diplotaxodon* species using genome scans and three independent candidate outlier detection approaches. We predict that, if the focal species are indeed specialized to different water depths within the twilight zone of Lake Malawi, candidate genomic regions of increased interspecific divergence should contain genes relevant for ecological and physiological adaptations associated with differences in, for example, ambient light conditions, hydrostatic pressure, or the availability of dissolved oxygen. We propose that signatures of divergent selection in such genes can be interpreted as indirect evidence for a role of depth‐related stratification in maintaining reproductive isolation between these species. We further characterize genomic variants associated with the observed interspecific eye morphological differences, and likely candidate regions, including ecophysiologically relevant key genes, for adaptation to life at depth.

## Methods

### RAD DATA SAMPLING, DNA EXTRACTION, LIBRARY PREPARATION, AND ILLUMINA SEQUENCING

**Figure 1:**
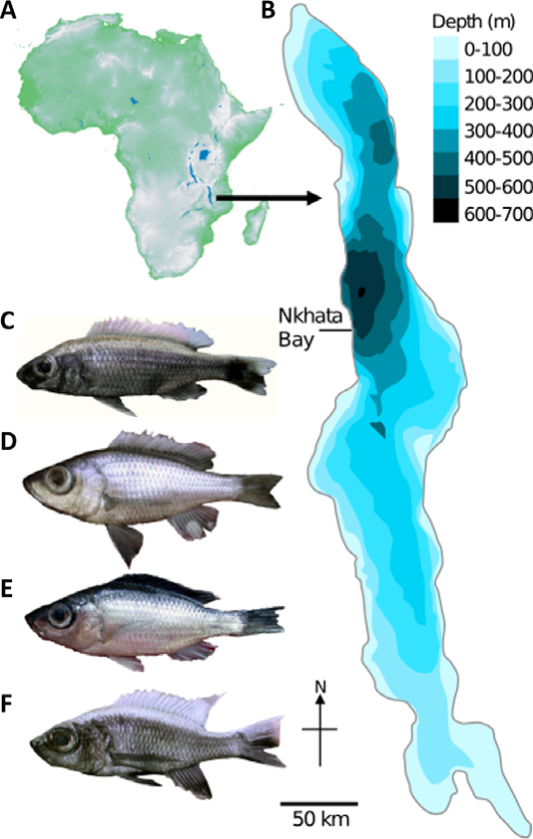
Maps of (A) Africa (topographic) and (B) Lake Malawi (bathymetric) indicating the sampling location Nkhata Bay. (C) *D*. “limnothrissa black pelvic”; (D) *D*. “macrops offshore”; (E) *D*. “macrops black dorsal”; (F) *D*. “macrops ngulube.”

A total of 40 individuals from four *Diplotaxodon* species were collected from Nkhata Bay (Fig. 1, Table S1), photographed, and fin clips stored in ethanol at −80°C. Genomic DNA was extracted from fin clips using the Qiagen DNAeasy Blood and Tissue Kit, according to manufacturer's instructions. DNA was quantified using PicoGreen fluorimetry (Quant‐iT PicoGreen Kit, Invitrogen) and quality checked on 0.8% agarose gels. Paired‐end RAD libraries were prepared by the NERC/NBAF facility at Edinburgh Genomics, following Baird et al. (2008) and Ogden et al. (2013). Genomic DNA was digested using *SbfI*, a barcoded RAD P1 adapter ligated, followed by sonic shearing, size selection, and ligation of P2 adapters. Libraries were PCR amplified, quantified, and sequenced in separate flow cells on an Illumina HiSeq 2000 platform with 100 bp, paired‐end chemistry.

### RAD DATA PROCESSING

Modules from the *Stacks* version 1.20 (Catchen et al. 2013) program suite were used for the initial processing of the RAD data as follows: Raw reads were demultiplexed based on their inline barcode and quality trimmed using the program *process_radtags*. Putative PCR duplicates were subsequently removed using the program *clone_filter* from *Stacks* version 1.20. The remaining reads for each individual were mapped to the draft genome of *Maylandia* ( = *Metriaclima*) *zebra* (Brawand et al. 2014), version MetZeb1.1_prescreen (downloaded from http://archive.broadinstitute.org/ftp/pub/assemblies/fish/M_zebra/MetZeb1.1_prescreen/M_zebra_v0.assembly.fasta; last accessed 23.12.2016) using *BWA* version 0.7.10‐r789 (Li and Durbin 2009), allowing for up to eight mismatches per read. Any reads mapping to more than one genomic location were removed from the dataset using a custom script (*split_sam.pl*) and the remaining reads were converted to BAM format using *SAMtools* version 0.1.19‐4428cd (Li et al. 2009). The program *pstacks* from *Stacks* version 1.20 was used to call SNPs using a multinomial‐based likelihood model for diploid organisms. We required a minimum support of five identical reads per individual to consider alleles in the SNP calling model. A global catalog of RAD tags was then built using *cstacks* from all individuals with at least 20,000 valid tags identified by *pstacks* (seven individuals with fewer valid tags were excluded from further analyses). The *populations* program from *Stacks* version 1.20 was used to calculate basic population genetics statistics, considering all sites with minimum read depth of 5× per individual, and to produce data files for downstream analyses (e.g., in plink format, structure format) for each population individually, for each pairwise population comparison, and across a global dataset of all populations. Analyses were limited to tags identified in all populations and in at least 80% of individuals per population, respectively. Maximum‐likelihood inference was performed on the concatenated RAD tags using *RAxML* version 8.2.4 (Stamatakis 2014), using the GTR + gamma model and 100 bootstrap replicates. SNP datasets to be used in downstream analyses were limited to only a single SNP per RAD tag (*–write_single_snp* option in *populations*) to reduce the effects of linkage. We furthermore excluded any loci with global minor allele counts of one, from any analyses. Further conversions to file formats not supported by *populations* were performed by *PGDSpider* version 2.0.7.3 (Lischer and Excoffier 2012) or by custom scripts. Principal component analysis (PCA) and discriminant analysis of principal components (DAPC) (Jombart et al. 2010) was performed using the *Adegenet* package in R (Jombart and Ahmed 2011).

### IDENTIFICATION OF PUTATIVE CANDIDATE LOCI UNDER SELECTION

Three independent approaches to outlier detection were applied: (1) pairwise *F_ST_* values were calculated by *populations* (Catchen et al. 2013). Significance of *F_ST_* values was assessed by comparing the kernel‐smoothed average across a sliding window (window size 50 kb) centered on a particular SNP against a genome‐wide average (empirical null distribution) that was obtained via bootstrap resampling (1,000,000 replicates) from loci of the entire dataset. Loci centered in windows with *P* < 5 × 10^−5^ in at least one pairwise comparison were considered candidate loci under selection identified by *Stacks*. (2) The Bayesian approach to outlier loci detection implemented in *Bayescan* version 2.1 (Foll and Gaggiotti 2008), which is based on decomposing population *F_ST_* coefficients into population‐ and locus‐specific variation using logistic regressions, was applied to pairwise, as well as a global, SNP datasets. In all Bayesian analyses, prior odds for the neutral model were set to 10, with the remaining parameters set to default. A false discovery rate (FDR) threshold of 0.05 was applied and loci with α < 0.05 in at least one pairwise comparison or the global run were considered candidate loci identified by *Bayescan*. (3) *Bayenv* version 2 (Coop et al. 2010) was used to identify candidate outliers based on the global SNP dataset obtained for all four populations. *Bayenv* accounts for population history by incorporating a covariance matrix of population allele frequencies and for differences in sample size among populations (Coop et al. 2010). Ten independent covariance matrices were constructed for sets of 5,000 SNPs randomly selected from the global dataset as represented in a vcf file produced by *populations*, using a custom script (*vcf_2_div.py*). In brief, for each random set covariance matrices were obtained by running *Bayenv* for 100,000 iterations. Convergence of covariance matrices was assessed visually in R (R Development Core Team 2014) and a final covariance matrix was obtained by averaging across the 10 independent runs. Using this matrix to account for population history *Bayenv* was then run for 20 independent replicates with 1,000,000 iterations each on a set of SNPs present in at least five individuals in each population. Candidate loci under selection were identified based on the statistic *xTx* (note that *xTx* is a population differentiation statistic and not a measure of correlation with environmental factors as described below) generated by *Bayenv* as follows: for each run we ranked loci based on their respective value for *xTx*, and calculated a rank statistic similar to the empirical *P*‐value approach used by Hancock et al. (2011). In our approach the highest ranking SNP would be assigned the highest relative rank of 1, whereas the lowest ranking SNP would be assigned a rank of 1/N, with Nbeing the total number of SNPs in the dataset. For each SNP we then calculate the average relative rank (ARR) and standard deviation across the 20 independent *Bayenv* runs. Loci yielding an ARR in the 95th percentile were considered candidate loci under selection identified by *Bayenv*. We then applied a Gaussian weighting function to generate a kernel‐smoothed moving average of the transformed rank statistic for each polymorphic site (based on 100 kb sliding windows centered at each SNP) on scaffolds that contained loci yielding ARR in the 95th percentile. To test for statistical significance of windows, we applied a bootstrap resampling procedure (10,000 permutations). In each permutation new values for ARR were sampled, with replacement, from across the entire dataset and the smoothed statistic was calculated for each replicate set using the coordinates of the original SNP for the weighing function. For each window, centering on a particular locus, the data obtained via bootstrap resampling was used as empirical null distribution of the test statistic against which the original smoothed average ARR was compared to determine a *P*‐value. The approach we have implemented is similar to the method used by the *populations* program of the *stacks* software suite (Catchen et al. 2013). Loci that yielded an ARR in the 95th percentile and were centered in a window with *P* < 0.005 were considered candidate loci under selection identified by *Bayenv*.

Genomic candidate regions under selection were defined as ±50 kb windows up and downstream of candidate SNPs, supported by one, two, or three outlier detection approaches, respectively. Candidate regions of consecutive candidate SNPs were merged in case an overlap between the corresponding windows was detected.

### IDENTIFICATION OF SNPs CORRELATED WITH MORPHOLOGICAL DIFFERENCES BETWEEN POPULATIONS

The Bayesian linear model approach implemented in *Bayenv* (Coop et al. 2010) is frequently used to infer correlations between SNP allele frequencies and environmental variables. The method yields Bayes factors (BF), which are interpreted as the weight of evidence for a model in which an environmental factor is affecting the distribution of variants relative to a model in which environmental factors have no effect on the distribution of the variant (Hancock et al. 2011). The four *Diplotaxodon* populations analyzed in the current study have been previously identified to differ significantly in head morphological traits (Genner et al. 2007), which in turn are known to correlate with environmental variables in cichlids (Bouton et al. 2002). In particular, *Diplotaxodon* “limnothrissa black pelvic” has smaller eyes than the other species. We applied the *Bayenv* approach, using vertical and horizontal eye diameter (normalized by individual fish total length, TL) obtained previously for the focal populations (Genner et al. 2007), to identify SNPs correlated with eye morphological differences between populations and to characterize the genomic regions involved in regulating the observed morphological differences. Prior to *Bayenv* analyses, population averages were standardized by subtracting the global mean and dividing the result by the global standard deviation. Standardized population averages were then used as “environmental variables” in 20 independent runs of *Bayenv*, each using 1,000,000 iterations. Transformed rank statistics by means of ARRs of BFs were calculated across the 20 runs and kernel smoothed averages and *P*‐values were calculated as described above. Windows significant at the *P* < 0.001 level were considered candidate regions associated with eye morphological differences if they also contained at least one individual SNP locus yielding an ARR in the 95th percentile of the original distribution. The full *Bayenv* workflow and the subsequent analyses are made available as Jupyter notebooks in a dedicated Github repository.

### IDENTIFICATION OF GENE FUNCTION AND ENRICHMENT ANALYSES IN CANDIDATE GENOMIC REGIONS UNDER SELECTION

Gene models for *M. zebra* (Brawand et al. 2014) were obtained from the Broad Institute (downloaded from http://ftp://ftp.broadinstitute.org/pub/vgb/cichlids/Annotation/Protein_coding/; last accessed 23.12.2016). Peptide sequences were subjected to a similarity search against a custom build of the NCBI's non‐redundant (nr) protein database (restricted to Metazoan proteins) using BLAST (Altschul et al. 1997) and screened for known domains using *InterProScan* 5.8‐49.0 (Quevillon et al. 2005). Results from these analyses were reconciled in *Blast2GO* version 3 (Conesa and Götz 2008) to obtain putative functional annotation including Gene Ontology (GO) terms (Ashburner et al. 2000) for *M. zebra* gene models, where possible. GO term enrichment analyses using Fisher's exact tests with multiple testing correction of FDR (Benjamini & Hochberg 1995), as implemented in *Blast2GO* version 3 (Conesa and Götz 2008), were performed for gene complements in genomic candidate regions supported by one, two, or three candidate outlier approaches, respectively.

### WHOLE GENOME RESEQUENCING DATA

Following up on our initial results, we selected the genes coding for rhodopsin, phakinin, and melanopsin, as candidate genes for visual adaptations, as well as the genes coding for haemoglobin subunits alpha and beta, potentially involved in physiological adaptations to deepwater conditions. We included a third haemoglobin subunit gene into our analyses that we manually identified in the candidate region, based on homology with the tilapia *Oreochromis niloticus*. This gene was not annotated in the reference genome version that we used for our analyses, presumably because it contained some missing data in the second exon and intron, but was added in later versions. We downloaded data mapping to scaffolds 12, 81, and 215 (where the candidate genes are located) from the Cichlid Diversity Sequencing project (SRA accession: PRJEB1254). These data were not collected specifically for our study, but there was good overlap in *Diplotaxodon* taxon sampling (three out of our four species covered; total of six), and so were used opportunistically to examine the candidate genes highlighted by our analyses in more detail with respect to putatively functionally relevant nucleotide polymorphisms in *Diplotaxodon* as well as the greater Lake Malawi cichlid flock. Data processing and variant calling was carried out as in Malinsky et al. (2015).

### IDENTIFICATION OF POSITIVE SELECTION IN CANDIDATE GENES

Candidate genes (*Rh1*, haemoglobin subunits alpha and beta, *BFSP2*, *OPN4*, *TMX3*, and *XFIN*) were tested for signatures of positive selection based on dN/dS ratios using the program *CODEML* of the *PAML* package version 4.8 (Yang 2007). Tests for selection on each gene were performed using data from 49 individuals across as many Lake Malawi species extracted from the whole genome resequencing data, including six species of *Diplotaxodon*, and *Pallidochromis tokolosh*. Heterozygous nucleotide positions in individuals were represented by the appropriate IUPAC nucleotide ambiguity codes. Phylogenetic relationships across the species were first inferred using *RAxML* version 8.2.4 (Stamatakis 2014) based on nucleotide data across the scaffolds 12, 81, and 215, and *CODEML* analyses were repeated for each topology that was recovered, to assure robustness of inference. We performed likelihood ratio tests (LRT) between the log‐likelihoods of the site models M1a (neutral) and M2a (positive selection) in the candidate genes to infer significance. If found significant at α < 0.05, the Bayes empirical Bayes (BEB) method (Yang et al. 2005) was applied to identify amino acid sites under positive selection.

## Results

### POPULATION STRUCTURE AND GENOME‐WIDE INTERSPECIFIC DIVERGENCE

**Figure 2:**
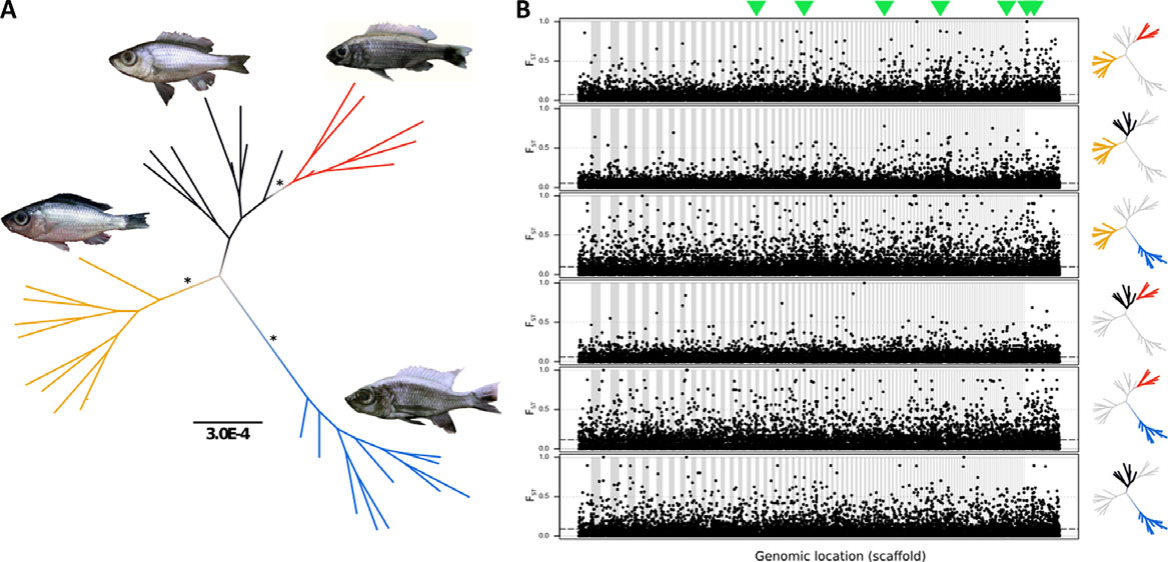
(A) Maximum‐likelihood tree (unrooted) of phylogenomic structure among *Diplotaxodon* species. Yellow—*D*. “macrops black dorsal”; red—*D*. “limnothrissa black pelvic”; black—*D*. “macrops offshore”; blue—*D*. “macrops ngulube.” Asterisks indicate >95% bootstrap branch support. Scale bar indicates genetic divergence (nucleotide divergence per site). (B) Genome‐wide pattern of pairwise divergence between populations. Dots represent pairwise *F_ST_* values for SNPs as calculated by *populations* (Catchen et al. 2013). Only scaffolds with minimum length of 100 kb containing a minimum of 10 SNPs are displayed. Individual scaffold boundaries are indicated by alternate white/grey background. Dashed lines indicate the global pairwise *F_ST_* average. Highlighted groups in the phylogenetic trees on the right‐hand side indicate the population pairs. Green arrow heads on top of the figure indicate locations of candidate regions supported by all three candidate outlier approaches (see Fig. S3).

After stringent filtering, the RAD data comprised 11,786 RAD tags that were each represented in at least 80% of individuals (minimum of 5) in each of the four populations (see Fig. 1, Table S1). Three methods confirmed that these are indeed four different species: maximum‐likelihood inference based on a concatenated alignment of these RAD tags (total alignment length 1,053,675 bp) (Fig. 2A). Branches separating species consistently receive high statistical support (bootstrap >95%). *D*. “macrops black dorsal”, *D*. “limnothrissa black pelvic”, and *D*. “macrops ngulube” were each reciprocally monophyletic, whereas *D*. “macrops offshore” was apparently paraphyletic with respect to *D*. “limnothrissa black pelvic.” PCA based on 11,786 SNPs (using only one SNP per RAD tag) confirmed strong population structure, with putative conspecific individuals grouping into distinct, nonoverlapping clusters along the first two principal components (Fig. S1). DAPC consistently assigned individuals with putative conspecifics (cluster assignment probability for all individuals 100%, *k* = 4, Fig. S2). Interspecific genome‐wide divergence (see Table S2) ranged from *F_ST_* = 0.05 (*D*. “limnothrissa black pelvic” vs. *D*. “macrops offshore”) to *F_ST_* = 0.09 (*D*. “limnothrissa black pelvic” vs. *D*. “macrops ngulube”). Figure 2B illustrates genome‐wide patterns of *F_ST_* divergence between species.

### CANDIDATE GENOMIC REGIONS UNDER SELECTION

We applied three distinct outlier detection approaches to identify candidate loci under positive selection between *Diplotaxodon* species. Together the three methods highlighted 242 loci (2.1% of total), distributed across 103 genomic scaffolds of the *M. zebra* reference genome version 1.1 (Brawand et al. 2014). The number of loci highlighted by individual methods ranged from 125 (1.1% of total, *Stacks*) to 96 (0.8% of total, *Bayenv*). Of the total candidate loci, 189 (77.8%), 41 (16.9%), and 13 (5.3%) were highlighted by one, two, or all three methods (see Fig. S3), and these were assigned to 134, 26, and eight candidate regions (defined as ±50 kb windows up‐ and downstream of candidate locus; overlapping candidate regions of consecutive SNPs were merged) on 103, 26, and eight genomic scaffolds, respectively (Table S3 summarizes the location and characterizes the gene complements associated with the candidate regions on the 103 genomic scaffolds).

GO enrichment analyses of genes in the candidate regions under divergent selection highlighted GO terms associated with sensory perception (e.g., axon extension, neuron development) and photoreceptor development (dynactin complex), embryonic development and morphogenesis (fibroblast growth factor receptor signalling pathway, positive regulation of cell proliferation, developmental growth involved in morphogenesis), and oxygen binding/transport (haemoglobin complex, oxygen transport/binding, gas transport, hem binding). Specific candidate genes under divergent selection associated with visual perception and signal transduction include *ACTR1B* (beta centractin), *Rh1* (rhodopsin), *PPIase* (peptidyl‐prolyl cis‐trans isomerase), *SGPP1* (sphingosine‐phosphate 1 phosphatase 1), and *DENND4B* (see Tables S3 for full gene complements). The phosphatase coded by *SGPP1* catalyzes the degradation of sphingosine‐1‐phosphate, a key regulator in photoreceptor development (Miranda et al. 2009; Rotstein et al. 2010). With respect to putative physiological adaptations to deepwater environments, a genomic region centered on a cluster of three haemoglobin subunit genes, HBA (haemoglobin subunit alpha, 2×) and HBB1 (haemoglobin subunit beta‐1), was highlighted by all three candidate outlier approaches.

**Figure 3:**
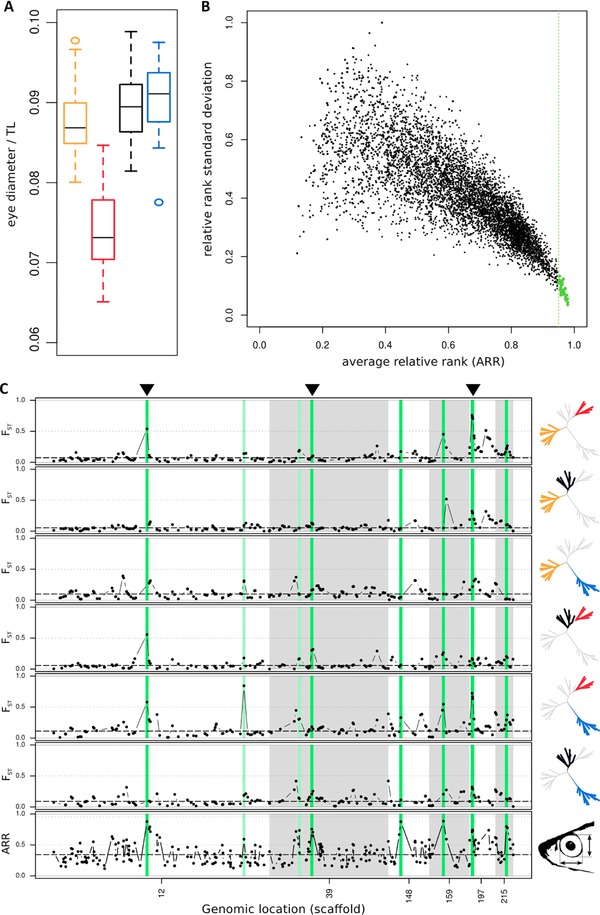
(A) *Diplotaxodon* eye diameter (normalized by total length of the fish, TL) across the four species. Yellow—*D*. “macrops black dorsal”; red—*D*. “limnothrissa black pelvic”; black—*D*. “macrops offshore”; blue—*D*. “macrops ngulube.” (B) Pope plot, summarizing correlations of population allele frequency with vertical eye diameter, inferred from 20 independent Bayenv runs. Dots illustrate per locus average relative rank (ARR) versus relative rank standard deviation. The green vertical line delimits the 95th ARR percentile. The 42 loci most consistently correlated with eye size (ARR ≥ 0.95) are shown in green. (C) Pairwise *F_ST_* divergence (top six panels) and global allele frequency correlations with interspecific vertical eye diameter differences (bottom panel). Displayed are the six scaffolds containing the most significantly correlated loci. Corresponding regions are highlighted in shades of green (light green—ARR ≥ 0.95; dark green—ARR ≥ 0.95, and smoothed ARR *P* < 0.001). Dots represent the kernel smoothed averages across 50 kb windows. Dashed lines indicate the genome wide average of F*_ST_*/ARR. Population pairs are indicated by the highlighted populations in the phylogenetic trees on the right‐hand side. Black arrowheads on top indicate regions that were also supported as candidate outlier loci by at least two of three independent outlier detection approaches. Table S5 summarizes the gene complements in highlighted regions.

Further candidate genes in regions under putative selection include genes central to craniofacial and eye morphogenesis, such as *ALX3* (aristaless‐like 3), *PXDN* (peroxidasin), *FOX* (Forkhead box transcription factor, 2×), *ZMIZ* (zinc finger miz domain containing protein, 2×), *RPGRIP1L* (retinitis pigmentosa GTPase regulator interacting protein 1, synonym Fantom), *MEIS2* (homeobox meis2 protein), *FGFR1* (fibroblast growth factor receptor 1), *SOX* (Sry box transcription factor, 2×), *DKK1* (Dickkopf1), *NEUCRIN* (Draxin), and *TCF7L1* (transcription factor 7‐like 1, formerly known as *TCF3*). *RPGRIP1L* has been shown to interact biochemically with *RPGR* (Retinitis Pigmentosa GTPase Regulator) (Khanna et al. 2009), which plays a central role in controlling access of both membrane and soluble proteins to the photoreceptor outer segment. Loss and mutations in *RPGR* have been associated with a range of retinal diseases in human patients, including a variant of cone dystrophy, characterized by progressive dysfunction of photopic (cone‐based) day vision with preservation of scotopic (rod‐based) night vision (Yang et al. 2002). *TCF7L1* is involved in the regulation of early embryonic craniofacial development via the Wnt/β‐catenin signalling pathway and is expressed during human embryonic eye development (Gaston‐Massuet et al. 2016). Generally the Tcf/Lef family of molecules mediate canonical Wnt signalling by regulating downstream target gene expression (Behrens et al. 1996). *TCF7L1* has been demonstrated to directly repress *SOX4 (*Gribble et al. 2009), which is expressed in early zebrafish eye development (Wen et al. 2015) and also plays an active role in Wnt signalling by stabilizing β‐catenin via a complex feedback loop (Bhattaram et al. 2014). Knockdown of *SOX4* in zebrafish resulted in structural malformations of the eye (Wen et al. 2015). *SOX2*, also affects Wnt signalling via feedback inhibition, and has been shown to crucially regulate retina formation in *Xenopus* (Agathocleous et al. 2009) and mice (Heavner et al. 2014). Mutations in the underlying gene have been associated with recessively inherited frontonasal malformation in humans (Twigg et al. 2009). Within the candidate regions identified by our analyses, we found cases of colocalization of genes potentially functionally relevant for deepwater adaptation, such as the close proximity of *ZMIZ*, *DKK1*, and *PPIase* (scaffold 197, Figs. 3C and S3), and *ACTR1B*, *FGFR1*, and *TCF7L1* (scaffold 45, see Fig. S3). *ACTR1B* is involved in regulating photoreceptor cell differentiation (Whited et al. 2004) and survival (Tsujikawa et al. 2007)*. ZMIZ* has been previously shown to directly interact in vitro with *Msx2*, an important regulatory element involved in skull (Wu et al. 1997) and specifically in eye morphogenesis (Foerst‐Potts and Sadler 1997). *DKK1* is an antagonistic inhibitor of the Wnt/β‐catenin signalling pathway, repeatedly implicated as a central mediator of craniofacial development in vertebrates (Brugmann et al. 2010; Liu et al. 2010), including cichlids (Loh et al. 2008; Parsons et al. 2014). In Lake Malawi cichlids an amino acid substitution in β‐catenin has been found to be associated with divergent jaw morphologies. *DKK1* inhibits the stabilization of β‐catenin by binding to *LRP6*. Colocalized with these two factors is a peptidyl‐prolyl cis‐trans isomerase (*PPIase*). Generally, *PPIases* are ubiquitous proteins, but in the context of this study it is worth noting that members of a *PPIase* subgroup (cyclophilins) have been shown to play a critical role in opsin biogenesis in *Drosophila (*Stamnes et al. 1991) and cattle (Ferreira et al. 1997). For some of the above‐mentioned candidate genes our outlier approaches have highlighted more than one paralog on separate genomic scaffolds. *ZMIZ*, *FOX*, and *SOX* transcription factors were present in two separate candidate regions, each (see Table S3).

### GENOMIC REGIONS ASSOCIATED WITH INTERSPECIFIC EYE SIZE VARIATION

Both vertical (Fig. 3A) and horizontal (Fig. S5) eye diameter differed significantly among the four *Diplotaxodon* species (ANOVA: vertical eye diameter *F*_3,171_ = 104.6, *P* < 0.001; horizontal eye diameter *F*_3,171_ = 127.7, *P* < 0.001), with *D. limnothrissa* eye diameters being significantly smaller in all pairwise comparisons (Tukey HSD: *P* < 0.001, Table S4). Enlargement of the eyes is often associated with adaptation to deepwater environments (Marshall and Marshall 1954; de Busserolles et al. 2013). We applied a Bayesian linear model approach using the observed eye size differences to identify genomic regions particularly associated with this phenotypic trait. These analyses highlighted 42 loci (0.4% of total, Fig. 3B), clustering into 37 genomic windows, with population allele frequencies highly correlated with interspecific eye diameter differences, as indicated by their ARR (ARR ≥ 0.95) across 20 independent *Bayenv* runs. Eight SNPs (0.07% of total) were found to be highly significantly associated with eye diameter using our most stringent filtering criteria (ARR ≥ 0.95 and smoothed ARR *P* < 0.001, all except the SNP on scaffold 39 were significant in both analyses, i.e., using vertical and horizontal diameter). This set of candidate SNPs clustered into six genomic regions (Fig. 3C). Three of these six genomic regions inferred as most significantly correlated with interspecific eye diameter variation, are also highlighted as candidate regions under selection by at least two of three independent outlier detection approaches (Fig. 3C).

GO enrichment analyses of the gene complements identified by the eye diameter informed analyses indicate significant overrepresentation of GO terms associated with, for example, “regulation of response to external stimulus” and “intermediate filament organization.” The corresponding genomic regions contain genes encoding for the photopigments, *Rh1*, *OPN4* (melanopsin), and *OPN5* (neuropsin), as well *SGPP1 and PPIase*, a structural eye lens protein (*BFSP2*), genes expressed during normal eye development (*TMX3, XFIN*), and central transcription factors regulating embryonic craniofacial development (*ZMIZ, DKK1, ALX3*). In particular *BFSP2*, *OPN4*, *TMX3*, and *XFIN* were found close together on scaffold 215. Table S5 details the gene complements in the genomic regions most significantly associated with eye size differences. Candidate genes contained in regions highlighted by both the eye morphology informed analysis and the general candidate outlier detection approaches include *Rh1*, *SGPP1*, *ZMIZ*, *DKK1*, *PPIase*, *ALX3*, and *RPGRIP1L* (Table S5).

### SIGNATURES OF SELECTION AND POTENTIALLY FUNCTIONAL SUBSTITUTIONS IN CANDIDATE GENES

Using genome‐level resequencing data obtained via the Malawi cichlid diversity sequencing project (NCBI Bioproject PRJEB1254), we examined nucleotide polymorphisms to identify potentially functional substitutions and tested for signatures of positive selection based on ratios of nonsynonymous to synonymous substitutions (dN/dS) in the genes coding for rhodopsin, phakinin, melanopsin, as well as haemoglobin subunits alpha and beta, all highlighted by the population level analyses described above. The resequencing data include 156 individuals from 78 haplochromine species from in and around Lake Malawi, including members of both the *D. limnothrissa* and *D. macrops* species complex (six *Diplotaxodon* species total). dN/dS ratio based tests revealed signatures of positive selection in genes coding for rhodopsin (χ^2^ = 221.72, *P* < 0.001), haemoglobin subunit alpha (χ^2^ = 147.47, *P* < 0.001), haemoglobin subunit beta (χ^2^ = 38.43, *P* < 0.001), phakinin (one of two predicted transcripts, χ^2^ = 21.46, *P* < 0.001), *TMX3* (χ^2^ = 6.62, *P* < 0.05), and *XFIN* (χ^2^ = 37.22, *P* < 0.001).

We identified a number of nonsynonymous substitutions, as well as intron‐, 5′‐ and 3′‐UTR polymorphisms, potentially relevant for differential visual and physiological adaptation in deepwater conditions (Table S6). Across *Diplotaxodon* species, we observed a total of eight nonsynonymous and three 3′‐UTR polymorphisms in the *Rh1* gene coding for rhodopsin. Three of the nonsynonymous polymorphisms involve variants so far restricted (private) to *Diplotaxodon*, with respect to the other taxa sampled so far from the greater Lake Malawi species flock. One further nonsynonymous variant appears private to the *Diplotaxodon‐Pallidochromis* lineage. Three of the amino acid residues affected by nonsynonymous substitutions (amino acid positions 83, 133, and 189) were previously considered likely to be involved in spectral tuning (see discussion for further details). We detected a total of 13, four, and two amino acids containing nonsynonymous polymorphisms in the haemoglobin subunit beta, and two subunit alpha genes, respectively, in the *Diplotaxodon* species. These included additional variants private to *Diplotaxodon*, while several others were observed at high frequency in *Diplotaxodon* and in additional taxa of a distinct deepwater lineage of benthic Lake Malawi cichlids, including *Alticorpus*, *Lethrinops*, and *Aulonocara*. Several of the affected residues are associated with changes in O_2_ affinity in the human haemoglobin homologues (Table S6). Further interspecific variation between *Diplotaxodon* species was observed in the intergenic region between haemoglobin subunit genes. Nonsynonymous variants private to *Diplotaxodon*, were also detected in the genes coding for phakinin and melanopsin, as well as *TMX3* and *XFIN*. In these genes, variation between *Diplotaxodon* species was most pronounced in intronic, 5′‐ and 3′‐UTR regions (Table S6).

## Discussion

In shallow‐water cichlids, species tend to segregate according to habitat, diet, and depth distributions. Thus, we predicted such resource partitioning is likely to be taking part in deepwater cichlids, and that genomic regions associated with differential depth adaptation should appear overrepresented in outlier loci. We first used RADseq data to identify genomic regions showing signals of strong differentiation between four species of deepwater *Dipotaxodon* species in Lake Malawi, and assumed that genes within 50 kb of outlier SNP loci were candidates for selection among *Diplotaxodon* species. GO enrichment analyses highlighted GO terms associated with sensory perception and photoreceptor development, embryonic development and morphogenesis, and oxygen binding/transport. We subsequently used genome‐level resequencing data and found signatures of positive selection in key candidate genes and patterns of SNP diversity in coding (and regulatory) regions consistent with ecological differences between *Diplotaxodon* species.

Our analyses indicate a role for key genomic regions containing genes that could be associated with adaptation to depth. A region centered on three haemoglobin genes shows a signal consistent with selection‐ and codon‐based tests confirmed that both haemoglobin alpha and beta subunit genes have been targets of selection. These genes are relevant for physiological adaptation to different water depths in two ways. The first is through enhancing the “Root effect,” a pH‐dependent decrease in oxygen‐carrying capacity of some fish haemoglobins. This facilitates O_2_ secretion into the swim bladder, the specialized organ found in most teleost fishes used to achieve neutral buoyancy in open water by regulating partial gas pressure in response to the ambient hydrostatic pressure. At the molecular level, such “Root haemoglobins” are characterized by a range of amino acid replacements in the globin α‐ and β‐chains, compared to “normal” haemoglobins (Perutz and Brunori 1982; Fago et al. 1997; Pelster 2001) and may have evolved independently a number of times (Berenbrink et al. 2005). The affected *Diplotaxodon* haemoglobin subunit genes fulfill the minimal structural requirements for Root haemoglobins as previously defined by (Mazzarella et al. 2006). The second way selection could act on this region is via haemoglobin O_2_ binding affinity (Thom et al. 2013). Given teleost fishes can differ in their tolerance toward low levels of dissolved oxygen, and the amount of dissolved oxygen in water typically decreases with depth, it is plausible that divergent selection is operating on oxygen tolerance in Lake Malawi (Mandic et al. 2009). Meromictic freshwater lakes exhibit complete oxygen depletion below a defined oxic‐anoxic boundary layer, which in Lake Malawi has been identified at ∼230 m water depth (Darwall et al. 2010). A number of the residues affected by nonsynonymous variants in *Diplotaxodon* (Table S6) have been associated with changes in oxygen affinity in the human haemoglobin homologues. Although both the Root effect and haemoglobin oxygen binding affinity have previously been predicted to be likely targets of natural selection (Sébert and Macdonald 1993; Mandic et al. 2009), few studies have to date been able to demonstrate selection acting on haemoglobin genes. Selection for high O_2_ affinity haemoglobin alleles was previously demonstrated in response to altitude‐related hypoxia in birds (Gou et al. 2005; Natarajan et al. 2015, 2016). In North Atlantic cod populations distinct distributions of functionally different haemoglobin variants, linked to polymorphisms in the haemoglobin beta subunit, were observed and proposed to reflect adaptations to differing temperature and oxygen regimes (Sick 1961; Andersen 2012). The exact effect of the observed nonsynonymous changes on the Root effect and/or the O_2_ affinity of *Diplotaxodon* haemoglobins needs to be determined experimentally, but the presence of the same changes in other unrelated deepwater Lake Malawi taxa such as deep‐benthic *Lethrinops* and *Alticorpus* spp. is consistent with adaptation to depth. Whether these changes have arisen multiple times independently, represent ancestral polymorphisms, or have been acquired through introgression, warrants further investigation. There is evidence of hybridization as a driver in adaptive radiation in the Lake Malawi species flock (Joyce et al. 2011; Genner and Turner 2012; Nichols et al. 2015) and these loci could allow a test of whether these deepwater adaptations have been retained after a hybridization event, and allowed subsequent radiation into this challenging habitat (Genner and Turner 2012). Star and colleagues described a polymorphism in the intergenic promoter region of the alpha–beta haemoglobin subunits in cod. They showed that promoter types differed in transcriptional activity and concluded that promoter‐mediated increased synthesis of globin variants could compensate for structurally mediated low oxygen affinity (Star et al. 2011). We observed variation in the intergenic regions (single nucleotide and indel variation) of haemoglobin subunit genes between *Diplotaxodon* species, which may indicate a role for transcriptional regulation of haemoglobin synthesis in differential adaptation also in this lineage.

Previous work has shown that the four *Diplotaxodon* species we studied differ significantly with respect to head and overall body morphology (Genner et al. 2007), and craniofacial variation in cichlids is frequently associated with trophic adaptations. Strongly differentiated cranial dimensions include eye size differences, and light conditions at different water depths are a likely selective driving force. We identified candidate genomic regions under selection that appear enriched for genes involved in the regulation of craniofacial development. Our outlier detection approaches highlight a number of Wnt factors, further supporting the central role of Wnt signalling for regulating cichlid craniofacial gene expression (Parsons et al. 2014). One genomic region in particular (scaffold 197, Fig. 3C) received strong support in all analyses, and contains a group of colocalized genes; namely *ZMIZ* (skull and eye morphogenesis), *DKK1* (vertebrate craniofacial development), and a *PPIase* (opsin biogenesis). The close proximity of *ZMIZ* and *DKK1* in particular may indicate they are inherited together as a craniofacial “supergene.”

The larger eye of species in the *Diplotaxodon macrops* “complex” relative to those in the *Diplotaxodon limnothrissa* “complex” is consistent with their presumed depth distributions (Figs. 3A and S5). The environment at depths of 50–200 m (Snoeks and Konings 2004) is depauperate of long wavelength light and dominated by shorter wavelength blue light as depth increases (Von der Emde et al. 2012). Our analyses consistently highlight a genomic region around the *Rh1* gene (scaffold 12, Fig. 3C), which codes for rhodopsin, the principal photopigment of retinal rod photoreceptors, central for scotopic vision under dim light conditions. Changes in both the coding sequence as well as in gene expression of opsins (Hofmann et al. 2009) may mediate visual performance across light environments, and the pivotal role of rhodopsin for visual adaptation in deepwater light environments has been confirmed by a number of studies (Sugawara et al. 2005; Sivasundar and Palumbi 2010; Wang et al. 2011; Nakamura et al. 2013; Shum et al. 2014). Codon‐based tests inferred a strong signature of positive selection acting on the rhodopsin gene. Whether the observed nonsynonymous variants (Table S6) result in functionally important shifts of the spectral sensitivity of the photopigment between *Diplotaxodon* species needs further investigation. However, it is worth noting that three of the 10 observed amino acid residues found affected by nonsynonymous polymorphisms identified in the current study (amino acid positions 83, 133, and 189) have previously been considered likely to be involved in spectral tuning, because of their close proximity to the chromophore or the chromophore‐binding pocket (Sugawara et al. 2005). Amino acid replacements at position 83, specifically, have been demonstrated experimentally to cause spectral shifts toward blue (Sugawara et al. 2005). A further four affected residues (166, 169, 297, 298) have also been implicated in a recent study of spectral shifts via mutations in the rhodopsin gene in an isolated East African crater lake (Malinsky et al. 2015).

In an attempt to characterize the genomic regions associated with the observed eye size differences, we adopted a Bayesian linear modeling approach, to identify loci at which population allele frequencies and eye size distributions between *Diplotaxodon* species are strongly correlated. The method was originally developed to identify candidate loci underlying local adaptation via correlation with environmental factors, but more generally was designed as a means of highlighting interesting loci and correlations that can be further explored by follow‐up studies (Coop et al. 2010). In addition to the genomic regions containing rhodopsin, as well as *ZMIZ* and *DKK1*, our eye size informed analyses have highlighted a region characterized by a high density of genes associated with eye development and vision (*BFSP2*, *OPN4*, *TMX3*, and *XFIN*). Exploration of whole genome resequencing data obtained for the larger Malawi flock revealed signatures of positive selection in *BFSP2*, *TMX3*, and *XFIN*, and nonsynonymous mutations private to the deepwater lineage in both the *BFSP2* and the *OPN4* gene. Variation between *Diplotaxodon* species is most pronounced in intronic and 5′‐UTR regions, which may imply a role of transcriptional regulation in differential adaptation of the species. The role these genes may play in visual adaptation is currently unknown. *XFIN* is expressed during the formation of the retina in *Xenopus* (Rijli et al. 1993) and deletion of the *TMX3* gene in humans has been linked to a genetic disease associated with retarded growth of the eye (Chao et al. 2010). *OPN4* codes for the opsin‐based photopigment melanopsin, which is central to a distinct photoreceptor class, the melanopsin retinal ganglion cells (mRGCs), that was discovered only relatively recently (Provencio et al. 2000). Initially mRGCs were shown to mediate so‐called nonimage forming visual responses (Panda et al. 2003), but more recent evidence suggests that mRGCs may contribute significantly to assessing brightness and play a more general role in supporting vision in mammals (Brown et al. 2010) and it may well play a role in deepwater (cichlid) vision. The lens of the vertebrate eye is composed of specialized epithelial lens fiber cells containing beaded filaments, specific cytoskeletal structures unique to the lens (Ramachandran et al. 2007). Phakinin is one of the two principal proteins forming the beaded filaments, so our results also suggest a role for eye lens structure in adaptation to dim, short wavelength light environments, an idea that has so far attracted very little attention. The role of beaded filaments in lens biology is not fully understood, but they appear essential in maintaining optical clarity and transparency of the lens (Blankenship et al. 2001; Oka et al. 2008). Mutations in *BFSP2* are associated with cataract formation, that is, a clouding of the eye lens in humans (Conley et al. 2000; Jakobs et al. 2000). Cataracts reduce the intensity and alter the chromaticity of light traveling through the lens (Delahunt et al. 2004), with potentially great effect on visual perception (Marmor 2006). After cataract surgery patients usually report a change in colour appearance associated with additional short wavelength light reaching the retina (Delahunt et al. 2004; Marmor 2006). The wider implication is that *BFSP2* may specifically regulate lens transparency for blue light and could be a key and previously unrecognized mediator for adaptation in dim, blue‐dominated light environments. The potential role of lens structure in adaptation is further confirmed by the highlighting of *FGFR1*, which is considered essential for lens fiber differentiation (Zhao et al. 2008) and *MEIS2*, a gene that directly regulates *Pax6* during vertebrate lens morphogenesis. The latter transcription factor has been demonstrated to play essential roles in lens differentiation and has previously been referred to as the “master control gene for morphogenesis and evolution of the eye” (Gehring 1996; Gehring and Ikeo 1999).

Depth and habitat segregation may be caused by a number of factors including competition for resources, breeding territories, or enemy‐free space (Schluter 2000). The four *Diplotaxodon* also differ in male nuptial colouration (Genner et al. 2007), which strongly implies an important role of visually informed mate choice, despite the twilight conditions they experience in their natural environments. Although many pelagic fish use countershading in background matching for camouflage (Ruxton et al. 2004), *D*. “macrops ngulube” and *D*. “macrops black dorsal” males have an opposite nuptial colouration, that may allow them to be more visible. The sensory drive hypothesis (Endler 1992) predicts that visual systems (along with signals and signalling behavior) will differentiate if local environments differ in their signal transmission quality. The observed interspecific differences in male nuptial colour in *Diplotaxodon* in combination with the inferred genomic footprints of sensory adaptation are consistent with the idea that reproductive isolation could arise as a consequence of sensory drive in deepwater systems.

In summary, our work has shown that the selection pressures within deepwater environments can be identified by their effects on the genome. We interpret finding clear signatures of divergent selection in genomic regions and genes relevant for depth‐related ecophysiological adaptation as evidence consistent with a role of depth specialization in maintaining reproductive isolation between sympatric *Diplotaxodon* species. In addition to genes previously associated with depth‐related spectral shifts (rhodopsin), we identify novel mechanisms of adaptation to deepwater conditions worth further investigation (e.g., Root effect haemoglobins and eye lens filament proteins). Outlier tests tend to highlight large‐effect loci with relatively simply genetic architecture (Wolf and Ellegren 2016), so these will be interesting possibilities with which to identify parallel evolution in other systems such as populations experiencing high‐altitude hypoxia or marine deepwater systems. Our results provide evidence of fixed genomic changes since deepwater conditions in Lake Malawi were attained possibly as recently as 75,000 years ago (Delvaux 1993; Ivory et al. 2016), and raise the intriguing possibility that hybridization between *Diplotaxodon* and the deep‐benthic clade of cichlids may have facilitated the latter's expansion into the twilight zone. Finally, we find that in candidate genomic regions under selection functionally associated genes are frequently in close proximity, such that cichlid adaptations and ecological differentiation may be facilitated by the presence of linked, coadapted gene complexes, or “supergenes.”

Associate Editor: Z. Gompert

## Supporting information


**Figure S1**. Principal component analysis of *Diplotaxodon* species, based on 11,786 SNPs.

**Figure S2**. DAPC of *Diplotaxodon* species, based on 11,786 SNPs.

**Figure S3**. Number and concordance of candidate loci highlighted by three independent outlier detection approaches.

**Figure S4**. Pairwise *FST* divergence at the six scaffolds (scaffold id on the *x* axis) containing loci highlighted as outliers by three independent detection approaches (none of which were informed by eye size differences).

**Figure S5**. *Diplotaxodon* interspecific vertical eye diameter variation (normalized by total length of the fish, TL).

**Table S1**. Metadata associated with samples used for RAD experiment.

**Table S2**. Pairwise global *F_ST_* between *Diplotaxodon* species as calculated by Stacks.

**Table S3**. Genomic location (scaffold id), putative functional annotation of genes (if available), gene ID, and RADtag ids localized in candidate regions under selection.

**Table S4**. Tukey HSD pairwise comparison *P*‐values; vertical eye diameter—above diagonal; horizontal eye diameter—below diagonal.

**Table S5**. Genomic location (scaffold id), putative functional annotation of genes (if available), gene ID, and RADtag ids localized in candidate regions most significantly associated with interspecific eye size variation (candidate region defined as ±50 kb windows up‐ and downstream of significant locus).

**Table S6**. Details on nucleotide polymorphisms and amino acid changes in candidate genes in *Diplotaxodon* sp. and the greater Lake Malawi flock.

Supporting Information
