# Supplementary material for: The genomic basis of cichlid fish adaptation within the deepwater “twilight zone” of Lake Malawi
Source: Evol Lett. 2017 Aug 29;1(4):184–98. doi: 10.1002/evl3.20 (PMC6124600; doi:10.1002/evl3.20)
Supplement: Supplementary file 1 — Figure S1. Principal component analysis of Diplotaxodon species, based on 11,786 SNPs. [file EVL3-1-184-s001.pdf]

The genomic basis of cichlid fish adaptation within the deep water ‘twilight zone’ of Lake Malawi

Christoph Hahn[1,2]*, Martin J Genner[3], George F Turner[4], Domino A Joyce[1].

1 Evolutionary and Environmental Genomics Group (@EvoHull), School of Environmental Sciences, University of Hull, Hull HU5 7RX, UK.
2 Institute of Zoology, University of Graz, A-8010 Graz, Austria.
3 School of Biological Sciences, University of Bristol, Bristol Life Sciences Building, 24 Tyndall Avenue, Bristol. BS8 1TQ. UK.
4 School of Biological Sciences, Bangor University, Bangor, Gwynedd LL57 2UW, Wales, UK.

** corresponding author*

Supplementary Data

Supplementary Data including scripts and jupyter notebooks detailing all analyses are deposited in a dedicated Github repository:
https://github.com/HullUni-bioinformatics/Diplotaxodon_twilight_RAD

Supplementary Figures

Supplementary Figures are also available as high quality images for separate download at:
https://github.com/HullUni-bioinformatics/Diplotaxodon_twilight_RAD/tree/master/SUPPLEMENTARY_DATA/SUPPLEMENTARY_FIGURES

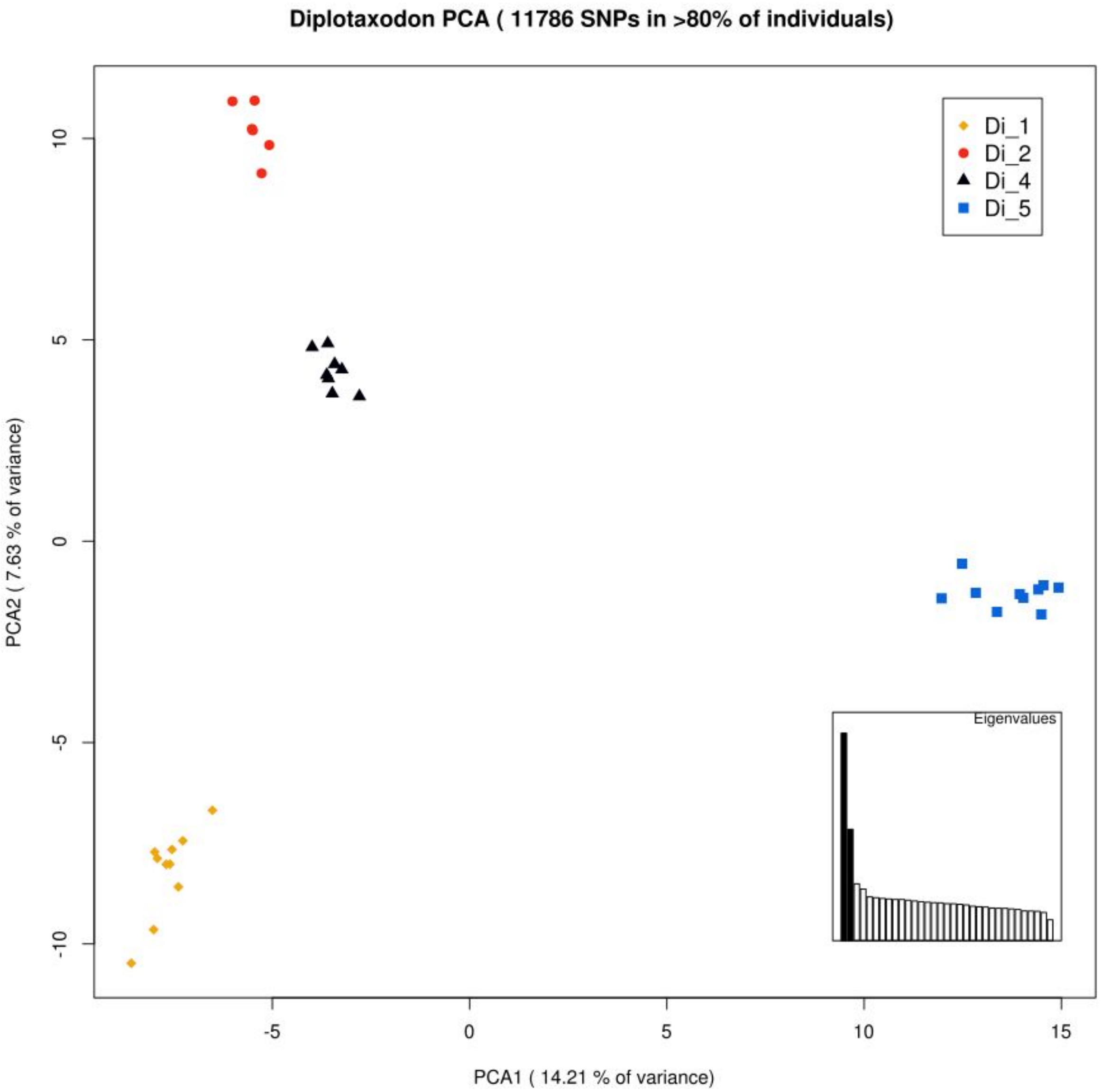

Figure S1. Principal component analysis of *Diplotaxodon* species, based on 11,786 SNPs. Yellow - *D*. 'macrops black dorsal'; red - *D*. 'limnothrissa black pelvic'; black - *D*. 'macrops offshore'; blue - *D*. 'macrops ngulube'.

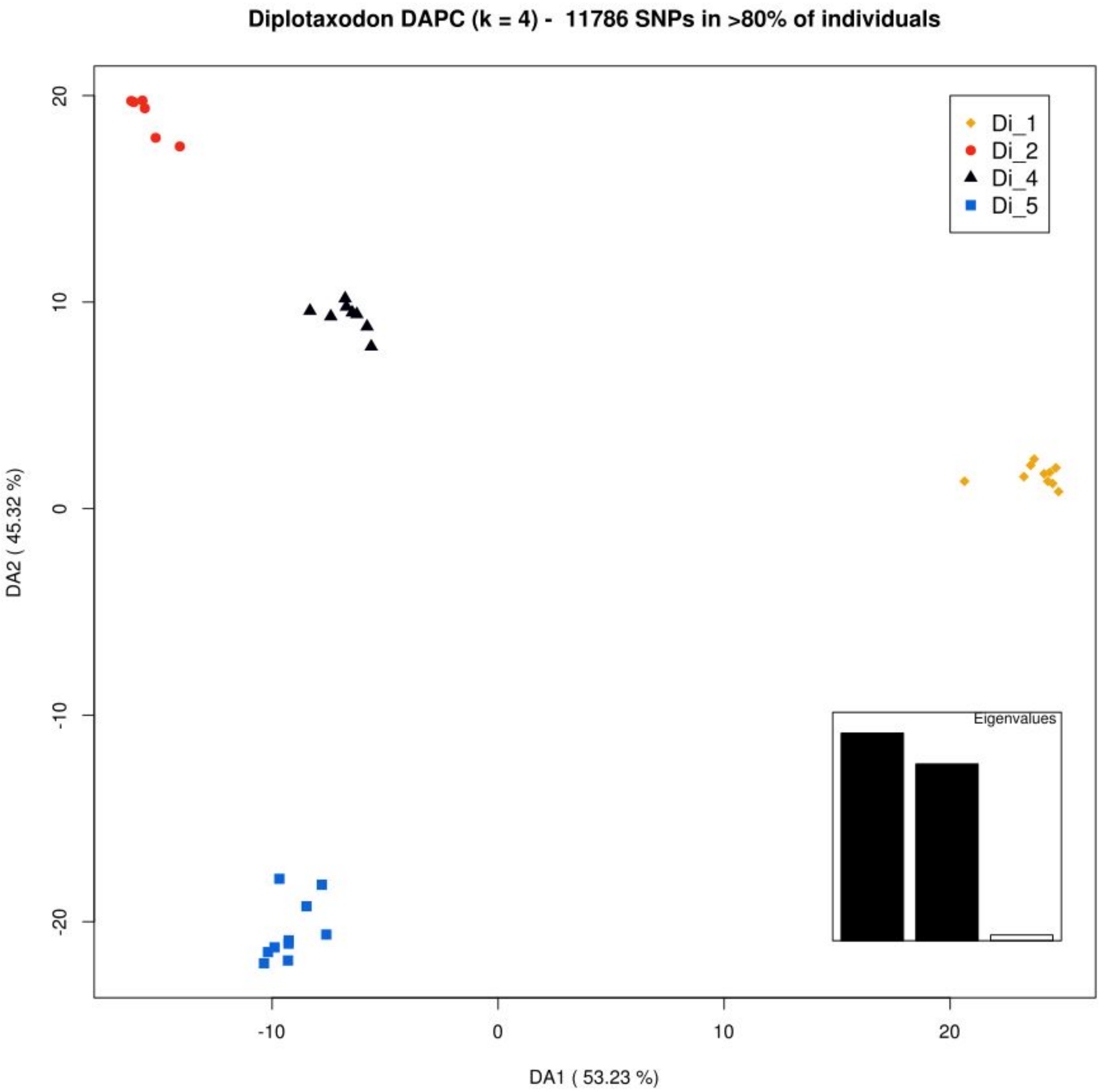

Figure S2. DAPC of *Diplotaxodon* species, based on 11,786 SNPs. Yellow - *D*. ‘macrops black dorsal’; red - *D*. ‘limnothrissa black pelvic’; black - *D*. ‘macrops offshore’; blue - *D*. ‘macrops ngulube’.

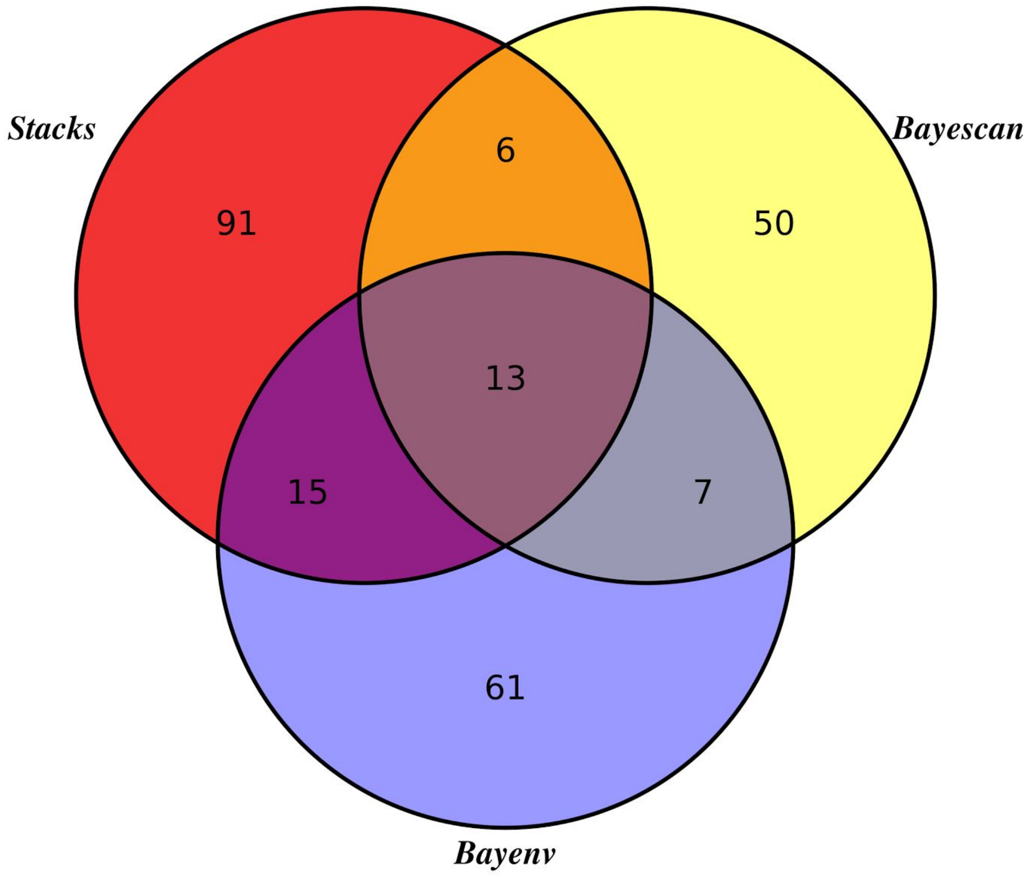

Figure S3. Number and concordance of candidate loci highlighted by three independent outlier detection approaches. Note that these analyses were not informed by eye size differences.

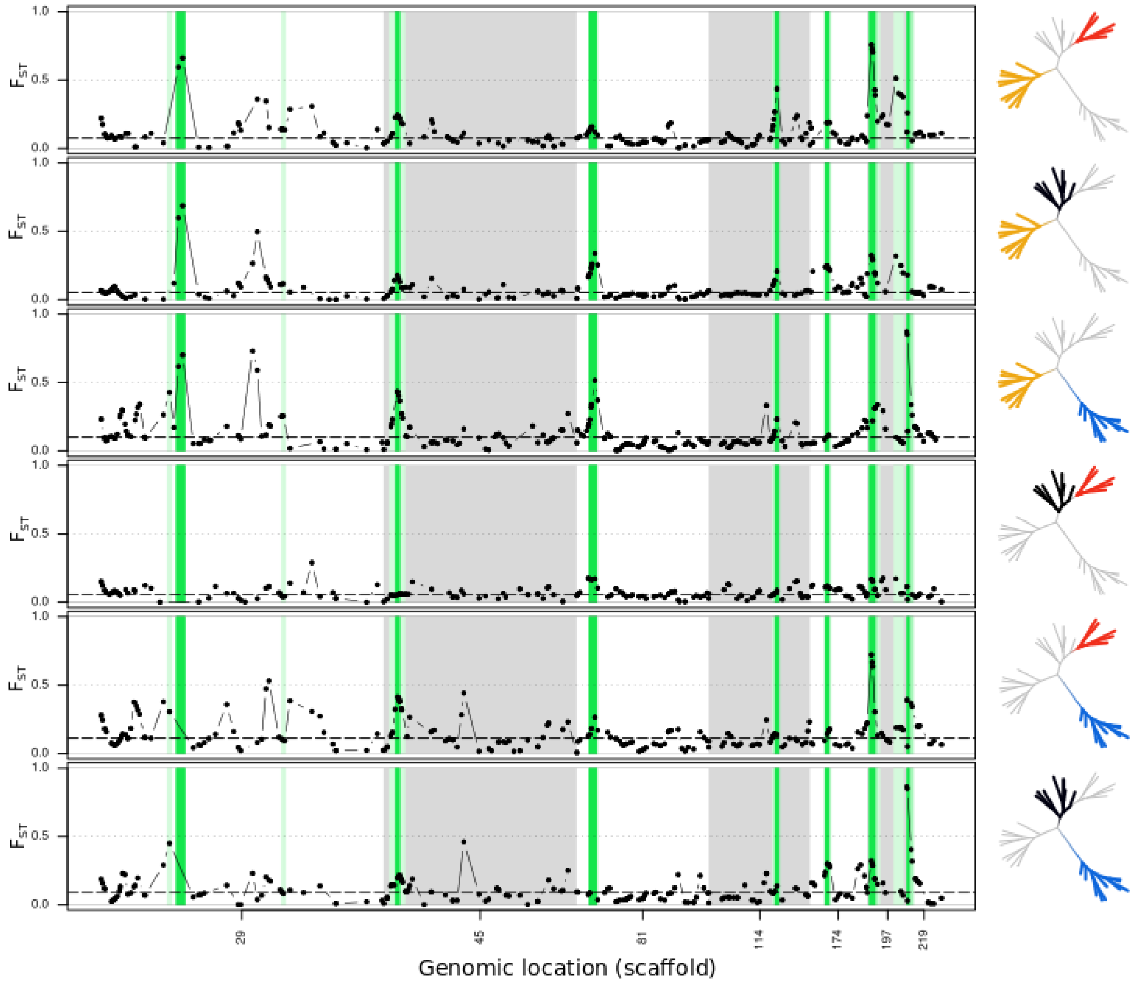

Figure S4. Pairwise F_{ST} divergence at the six scaffolds (scaffold id on the x axis) containing loci highlighted as outliers by three independent detection approaches (none of which were informed by eye size differences). Displayed are only scaffolds containing a minimum of 5 SNPs. Putative candidate regions are highlighted in shades of green. Dark green regions indicate support by three approaches (see table S3 for summary of gene complements in highlighted regions). Dots represent the kernel smoothed averages across 50kb windows as inferred by *populations*. Dashed lines indicate the genome wide F_{ST} average. Population pairs are indicated by the highlighted regions in the phylogenetic trees on the right hand side, *Diplotaxodon* species. Yellow - *D*. 'macrops black dorsal'; red - *D*. 'limnothrissa black pelvic'; black - *D*. 'macrops offshore'; blue - *D*. 'macrops ngulube'.

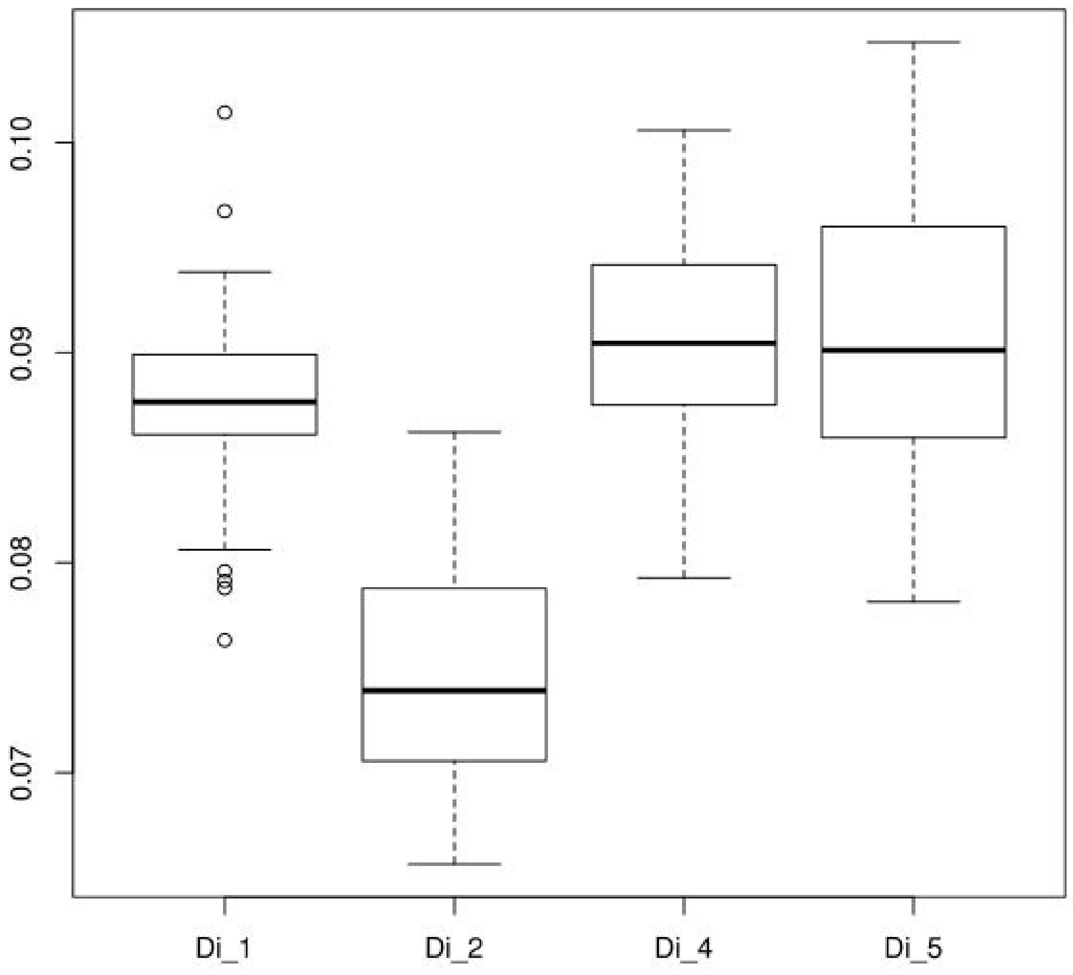

Figure S5. *Diplotaxodon* interspecific vertical eye diameter variation (normalized by total length of the fish, TL).

Supplementary tables

Supplementary tables are deposited on Github: https://github.com/HullUni-bioinformatics/Diplotaxodon_twilight_RAD/tree/master/SUPPLEMENTARY_DATA/SUPPLEMENTARY_TABLES

Table S1. Metadata associated with samples used for RAD experiment.
Table S2. Pairwise global Fst between Diplotaxodon species as calculated by Stacks.
Table S3. Genomic location (scaffold id), putative functional annotation of genes (if available), gene ID and RADtag ids localized in candidate regions under selection. Numbers in parenthesis after the RADtag id describe the number of independent outlier detection approaches supporting the respective tag, followed by the same tag's ARR and smoothed ARR level of significance (NS: > 0.05; *: 0.05-0.005; **: 0.005-0.001; ***: <0.001), inferred by the eye morphology informed analysis.
Table S4. TukeyHSD pairwise comparison p-values; Vertical eye diameter - above diagonal; horizontal eye diameter - below diagonal.
Table S5. Genomic location (scaffold id), putative functional annotation of genes (if available), gene ID and RADtag ids localized in candidate regions most significantly associated with interspecific eye size variation (candidate region defined as +-50 kb windows up and downstream of significant locus). Numbers in parenthesis after the RADtag id describe the tag's ARR and smoothed ARR level of significance (NS: >0.05; *: 0.05-0.005; **: 0.005-0.001; ***: <0.001).
Table S6. Details on nucleotid polymorphisms and amino acid changes in candidate genes in Diplotaxodon sp. and the greater Lake Malawi flock.

Further supplementary files are deposited on Github: https://github.com/HullUni-bioinformatics/Diplotaxodon_twilight_RAD/tree/master/SUPPLEMENTARY_DATA/MISC
