# Supplementary figures and images for: The genomic basis of cichlid fish adaptation within the deepwater “twilight zone” of Lake Malawi
Source: Evol Lett. 2017 Aug 29;1(4):184–98. doi: 10.1002/evl3.20 (PMC6124600; doi:10.1002/evl3.20)

**Diplotaxodon PCA ( 11786 SNPs in >80% of individuals)**

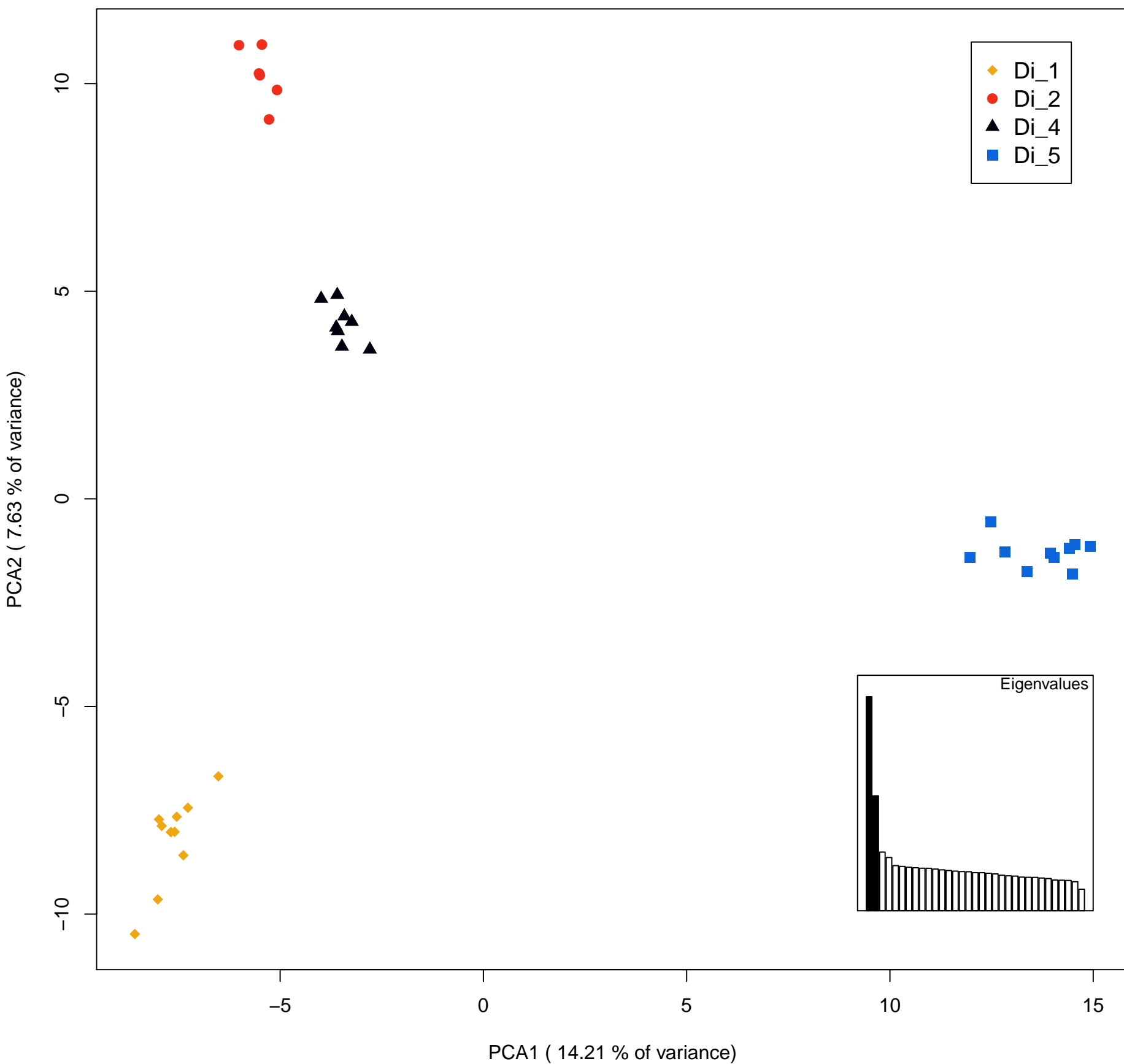

Supplement: Supplementary file 2 — Figure S2. DAPC of Diplotaxodon species, based on 11,786 SNPs. [file EVL3-1-184-s002.pdf]

Diplotaxodon DAPC ( 11786 SNPs in >80% of individuals) – k = 4

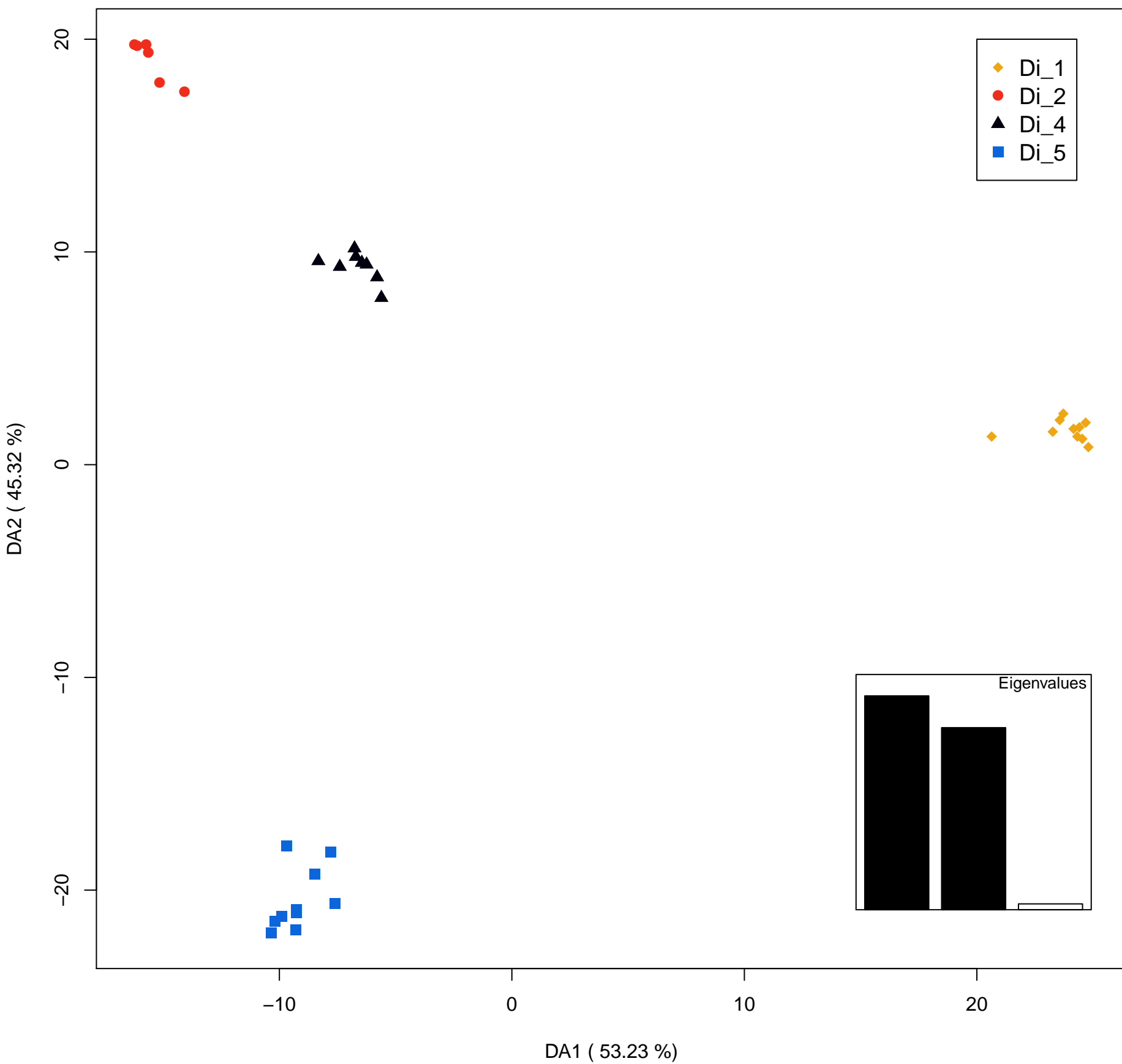

Supplement: Supplementary file 3 — Figure S3. Number and concordance of candidate loci highlighted by three independent outlier detection approaches. [file EVL3-1-184-s003.pdf]
