# Supplementary material for: The genomic basis of cichlid fish adaptation within the deepwater “twilight zone” of Lake Malawi
Source: Evol Lett. 2017 Aug 29;1(4):184–98. doi: 10.1002/evl3.20 (PMC6124600; doi:10.1002/evl3.20)
Supplement: Supplementary file 4 — Figure S4. Pairwise FST divergence at the six scaffolds (scaffold id on the x axis) containing loci highlighted as outliers by three independent detection approaches (none of which were informed by eye size differences). [file EVL3-1-184-s004.pdf]

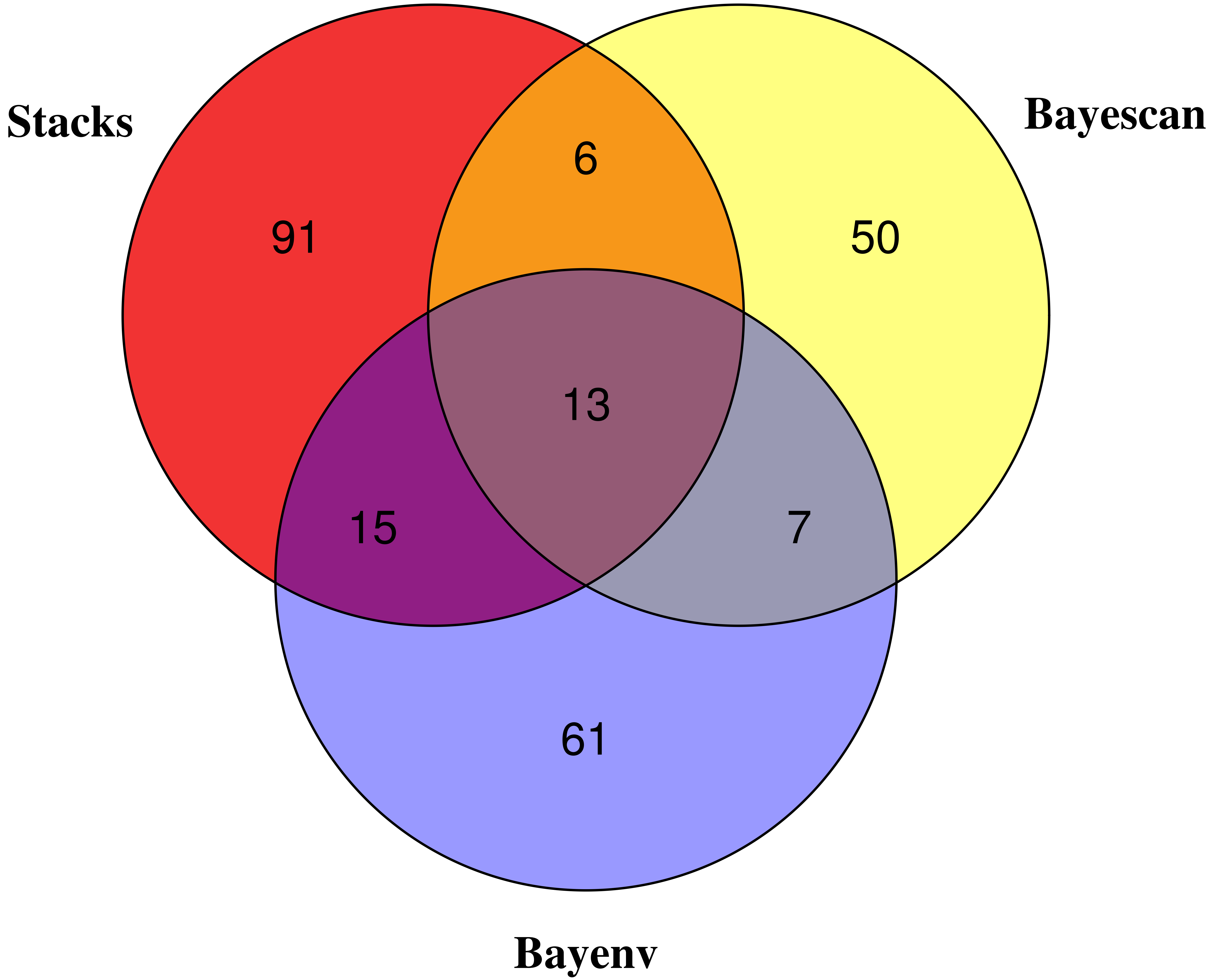

Stacks
Bayescan
6
91
50
13
15
7
61
Bayenv
